# Salient Object Detection by LTP Texture Characterization on Opposing Color Pairs under SLICO Superpixel Constraint

**DOI:** 10.3390/jimaging8040110

**Published:** 2022-04-13

**Authors:** Didier Ndayikengurukiye, Max Mignotte

**Affiliations:** Département d’Informatique et de Recherche Opérationnelles, Université de Montréal, Montréal, QC H3T 1J4, Canada; mignotte@iro.umontreal.ca

**Keywords:** color imaging, visual attention, salient object detection, color textures, local ternary pattern, fastmap

## Abstract

The effortless detection of salient objects by humans has been the subject of research in several fields, including computer vision, as it has many applications. However, salient object detection remains a challenge for many computer models dealing with color and textured images. Most of them process color and texture *separately* and therefore implicitly consider them as independent features which is not the case in reality. Herein, we propose a novel and efficient strategy, through a simple model, almost without internal parameters, which generates a robust saliency map for a natural image. This strategy consists of integrating color information into local textural patterns to characterize a color micro-texture. It is the simple, yet powerful LTP (Local Ternary Patterns) texture descriptor applied to opposing color pairs of a color space that allows us to achieve this end. Each color micro-texture is represented by a vector whose components are from a superpixel obtained by the SLICO (Simple Linear Iterative Clustering with zero parameter) algorithm, which is simple, fast and exhibits state-of-the-art boundary adherence. The degree of dissimilarity between each pair of color micro-textures is computed by the FastMap method, a fast version of MDS (Multi-dimensional Scaling) that considers the color micro-textures’ non-linearity while preserving their distances. These degrees of dissimilarity give us an intermediate saliency map for each RGB (Red–Green–Blue), HSL (Hue–Saturation–Luminance), LUV (L for luminance, U and V represent chromaticity values) and CMY (Cyan–Magenta–Yellow) color space. The final saliency map is their combination to take advantage of the strength of each of them. The MAE (Mean Absolute Error), MSE (Mean Squared Error) and *F*${}_{\beta }$ measures of our saliency maps, on the five most used datasets show that our model outperformed several state-of-the-art models. Being simple and efficient, our model could be combined with classic models using color contrast for a better performance.

## 1. Introduction

Humans—or animals in general—have a visual system endowed with attentional mechanisms. These mechanisms allow the human visual system (HVS) to select from the large amount of information received that which is relevant and to process in detail only the relevant aspects [1]. This phenomenon is called visual attention. This mobilization of resources for the processing of only a part of whole information allows its rapid processing. Thus the gaze is quickly directed towards certain objects of interest. For living beings, this can sometimes be vital as they can decide whether they are facing prey or a predator [2].

Visual attention is carried out in two ways, namely *bottom-up attention* and *top-down attention* [3]. *Bottom-up attention* is a process which is fast, automatic, involuntary and directed by the image properties almost exclusively [1]. The *top-down attention* is a slower, voluntary mechanism directed by cognitive phenomena such as knowledge, expectations, rewards, and current goals [4]. In this work, we focus on the *bottom-up attentional mechanism* which is image-based.

Visual attention has been the subject of several research works in the fields of cognitive psychology [5,6] and neuroscience [7], to name a few. Computer vision researchers have also used the advances in cognitive psychology and neuroscience to set up computational visual saliency models that exploit this ability of the human visual system to quickly and efficiently understand an image or a scene. Thus, many computational visual saliency models have been proposed and are mainly subdivided into two categories: conventional models (e.g., Yan et al. model [8]) and deep learning models (e.g., Gupta et al. model [9]). For more details, most of the models can be found in these works [10,11,12]).

Computational visual saliency models have several applications such as image/video compression [13], image correction [14], iconography artwork analysis [15], image retrieval [16], advertisements optimization [17], aesthetics assessment [18], image quality assessment [19], image retargeting [20], image montage [21], image collage [22], object recognition, tracking, and detection [23], to name but a few.

Computational visual saliency models are oriented to either eye fixation prediction or salient object segmentation or detection. The latter is the subject of this work. Salient object detection is materialized with saliency maps. A saliency map is represented by a grayscale image in which an image region must be whiter as it differs significantly from the rest of the image in terms of shape, set of shapes with a color, mixture of colors, movement, or a discriminating texture or generally any attribute perceived by the human visual system.

Herein, we propose a simple and nearly parameter-free model which gives us an efficient saliency map for a natural image using a new strategy. The proposed model, contrary to classical salient detection methods, uses texture and color features in a way that integrates color in texture features using simple and efficient algorithms. Indeed, the *texture* is a ubiquitous phenomenon in natural images: images of mountains, trees, bushes, grass, sky, lakes, roads, buildings, and so forth appear as different types of texture (Haidekker [24] argues that *texture* and shape analysis are very powerful tools for extracting image information in an unsupervised manner. This author adds that the *texture* analysis has become a key step in the quantitative and unsupervised analysis of biomedical images [24]. Other authors, such as Knutsson and Granlund [25], Ojala et al. [26], agree that *texture* is an important feature for scene analysis of images. Knutsson and Granlund also claim that the presence of a *texture* somewhere in an image is more a rule than an exception. Thus, *texture* in the image has been shown to be of great importance for image segmentation, interpretation of scenes [27], in face recognition, facial expression recognition, face authentication, gender recognition, gait recognition and age estimation, to just name a few [28]). In addition, natural images are usually also color images and it is then important to take this factor into account as well. In our application, the color is taken into account and integrated in an original way, *via* the extraction of the textural characteristics made on the pairs of opposing color spaces.

Although there is much work relating to *texture*, there is no formal definition of *texture* [25]. There is also no agreement on a single technique for measuring texture [27,28]. Our model uses the LTP (*local ternary patterns*) [29] texture measurement technique. The LTP (local ternary patterns) is an extension of local binary pattern (LBP) with three code values instead of two for LBP. LBP is known to be a powerful texture descriptor [28,30]. Its main qualities are invariance against monotonic gray level changes and computational simplicity and its drawback is that it is sensitive to noise in uniform regions of the image. In contrast, LTP is more discriminant and less sensitive to noise in uniform regions. The LTP (*Local Ternary Patterns*) is therefore better suited to tackle our salience detection problem. Certainly, the presence in natural images of several patterns make the detection of salient objects complex. However, the model we propose does not just focus on the patterns in the image by processing them separately from the colors as most models do [31,32] but it takes into account both the presence in natural images of several patterns and color, not separately. This task of integrating color in texture features is accomplished through LTP (Local Ternary Patterns) applied to opposing color pairs of a given color space. The LTP describes the local textural patterns for a grayscale image through a code assigned to each pixel of the image by comparing it with its neighbours. When LTP is applied to an opposing color pair, the principle is similar to that used for a grayscale image. However, for LTP on an opposing color pair, the local textural patterns are obtained thanks to a code assigned to each pixel, but the value of the pixel of the first color of the pair is compared to the equivalents of its neighbours in the second color of the pair. The color is thus integrated to the local textural patterns. In this way, we characterize the color micro-textures of the image without separating the textures in the image and the colors in this same image. The color micro-textures’ boundaries correspond to the superpixel obtained thanks to the SLICO (Simple Linear Iterative Clustering with zero parameter) algorithm [33] which is faster and exhibits state-of-the-art boundary adherence. We would like to point out that there are other superpixels algorithms that have a good performance such as the AWkS algorithm [34]; however, we chose SLICO because it is fast and almost parameter-free. A feature vector representing the color micro-texture is obtained by the concatenation of the histograms of the superpixel (defining the micro-texture) of each opposing color pair. Each pixel was then characterized by a vector representing the color micro-texture to which it belongs. We then compared the color micro textures characterizing each pair of pixels of the image being processed thanks to the fast version of the MDS (multi-dimensional scaling) method *FastMap* [35]. This comparison permits us to capture the degree of a pixel’s uniqueness or a pixel’s rarity. The FastMap method will allow this capture while taking into account the non-linearities in the representation of each pixel. Finally, since there is no single color space suitable for color texture analysis [36], we combined the different maps generated by FastMap from different color spaces (see Section 3.1), such as RGB, HSL, LUV and CMY, to exploit each other’s strengths in the final saliency map.

Thus, the contribution of this work is twofold:

- we propose an unexplored approach to salient object detection. Indeed, our model *integrates* the color information into the texture whereas most of the models in the literature that use these two visual characteristics, namely color and texture, process them *separately* thus implicitly considering them as independent characteristics. Our model, on the other hand, allows us to compute saliency maps that take into account the interdependence of color and texture in an image as they are in reality;
- we also use the *FastMap* method which is conceptually both local and global allowing us to have a simple and efficient model whereas most of the models in the literature use either a local approach or a global approach and other models combine these approaches in salient object detection.

Our model highlights the interest in opposing colors for the salient object detection problem. In addition, this model could be combined and be complementary with more classical approaches using the contrast ratio. Moreover, our model can be parallelized (using the massively parallel processing power of GPUs: graphics processing units) by processing each opposing color pair in parallel.

The rest of this work is organized as follows: Section 2 presents some models related to this approach with an emphasis on the features used and how their dissimilarities are computed. Section 3 presents our model in detail. Section 4 describes the datasets used, our experimental results, the impact of the color integration in texture and the comparison of our model with state-of-the-art models. Section 5 discusses our results but also highlights the strength of our model related to our results. Section 6 concludes this work.

## 2. Related Work

Most authors define salient object detection as a capture of the uniqueness, distinctiveness, or rarity of a pixel, a superpixel, a patch, or a region of an image [11]. The problem of detecting salient objects is therefore to find the best characterization of the pixel, the patch or the superpixel and to find the best way to compare the different pixels (patch or superpixel) representation to obtain the best saliency maps. In this section, we present some models related to this work approach with an emphasis on the features used and how their dissimilarities are computed.

Thanks to studies in cognitive psychology and neuroscience, such as those by Treisman and Gelade [37], Wolfe et al. [6,38] and Koch and Ullman [7], the authors of the seminal work of Itti et al. [39]—oriented eye fixation prediction—chose as features: color, intensity and orientation. Frintrop et al. [40], adapting the Itti et al. model [39] for salient objects segmentation—or detection—chose color and intensity as features. In the two latter models, the authors used pyramids of Gaussian and center-surround differences to capture the distinctiveness of pixels.

The Achanta et al. model [41] and the histogram-based contrast (HC) model [42] used color in CIELab space to characterize a pixel. In the latter model, the pixel’s saliency is obtained using its color contrast to all other pixels in the image by measuring the distance between the pixel for which they are computing saliency and all other pixels in the image; this is coupled with a smoothing procedure to reduce quantization artifacts. The Achanta et al. model [41] computed a pixel’s saliency on three scales. For each scale, this saliency is computed as the Euclidean distance between the average color vectors of the inner region $R_{1}$ and that of the outer region $R_{2}$, both centered on that pixel mentioned above.

Joseph and Olugbara [43] used color histogram clustering to determine suitable homogeneous regions in image and compute each region saliency based on color contrast, spatial features, and center prior.

Guo and Zhang [44], in the phase spectrum of the Quaternion Fourier Transform model, represent each image’s pixel by a Quaternion that consists of color, intensity and a motion feature. A Quaternion Fourier Transform (QFT) is then applied to that representation of each pixel. After setting the module of the result of the QFT to 1 to keep only the phase spectrum in the frequency domain, this result is used to reconstruct the Quaternion in spatial space. The module of this reconstructed Quaternion is smoothed with a Gaussian filter and this then produces the spatio-temporal saliency map of their model. For static images the motion feature is set to zero.

Other models also take color and position as features to characterize a region or patch instead of a pixel [42,45,46]. They differ, however, in how they obtain the salience of a region or patch. Thus, the region-based contrast (RC) model [42] measured the region saliency as the contrast between this region and the other regions of the image. This contrast is also weighted depending on the spatial distance of this region relative to the other regions of the image.

In the Perazzi et al. model [45], contrast is measured by the uniqueness rate and the spatial distribution of small perceptually homogeneous regions. The uniqueness of a region is calculated as the sum of the Euclidean distances between its color and the color of each region weighted by a Gaussian function of their relative position. The spatial distribution of a region is given by the sum of the Euclidean distances between its position and the position of each region weighted by a Gaussian function of their relative color. The region saliency is a combination of its uniqueness and its spatial distribution. Finally, the saliency of each pixel in the image is a linear combination of the saliency of homogeneous regions. The weight for each region’s saliency of this sum is a Gaussian function of the Euclidean distances between the color of the pixel and the colors of the homogeneous regions and the Euclidean distances between its spatial position and theirs. In the Goferman et al. model [46], the dissimilarity between two patches is defined as directly proportional to the Euclidean distance between the colors of the two patches and inversely proportional to their relative position normalized to be between 0 and 1. The salience of a pixel at a given scale is then 1 minus the inverse of the exponential of the mean of the dissimilarity between the patch centered on this pixel and the patches which are more similar to it; the final saliency of the pixel being the average of the saliency of the different scales to which they add the context.

Some models focus on the patterns as features but they compute patterns separately from colors [31,32]. For example Margolin et al. [31] defined a salient object as consisting of pixels whose local neighborhood (region or patch) is distinctive in both color and pattern. The final saliency of their model is the product of the color and pattern distinctness weighted by a Gaussian to add a center-prior.

As Frintrop et al. [40] stated, most saliency systems use intensity and color features. They are differentiated by the feature extraction and the general structure of the models. They have in common the computation of the contrast relative to the features chosen since the salient objects are so because of the importance of their dissimilarities with their environment. However, models in the literature differ on how these dissimilarities are obtained. Even though there are many salient object detection models, the detection of salient objects remains a challenge [47].

The contribution of this work is twofold:

- we propose an unexplored approach to the detection of salient objects. Indeed, we use for the first time in the salient object detection, to our knowledge, the feature *color micro-texture* in which the *color* feature is integrated *algorithmically* into the local textural patterns for salient object detection. This is done by applying LTP (Local Ternary Patterns) to each of the opposing color pairs of a chosen color space. Thus, in salient object detection computation, we *integrate* the color information in the texture while most of the models in the literature which use these two visual features, namely color and texture, perform this computation *separately*;
- we also use the *FastMap* method which, conceptually, is both local and global while most of the models in the literature use either a local approach or a global approach and other models combine these approaches in saliency detection. *FastMap* can be seen as a nonlinear one-dimensional reduction of the micro-texture vector taken locally around each pixel with the interesting constraint that the (Euclidean) difference existing between each pair of (color) micro textural vectors (therefore centered on two pixels of the original image) is preserved in the reduced (one-dimensional) image and is represented (after reduction) by two gray levels separated by this same distance. After normalization, a saliency measure map (with range values between 0 and 1) is estimated in which lighter regions are more salient (higher relevance weight) and darker regions are less salient.

Most of the models in the literature use either a local approach or a global approach and other models combine these approaches in saliency detection.

The model we propose in this work is both simple and efficient while being almost parameter free. Being simple and being different from the classic salience detection models which use the color contrast strategy between a region and other regions of an image, our model could therefore be effectively combined with these models for a better performance. Moreover, by processing each opposing color pair in parallel, our model can be parallelized using the massively parallel processing power of GPUs (graphics processing units). In addition, it produces good results in comparison with the state-of-the-art models in [48] for the ECSSD, MSRA10K, DUT-OMRON, THUR15K and SED2 datasets.

## 3. Proposed Model

### 3.1. Introduction

In this work, we present a model that does not require any learning basis and that highlights the interest of color opposing for the salient object detection problem. The main idea of our model is to algorithmically integrate the color feature into the textural characteristics of the image and then to describe this vector of textural characteristics by an intensity histogram.

To incorporate the color into the texture description, we mainly relied on the opponent color theory. This theory states that the HVS interprets information about color by processing signals from the cone and rod cells in an antagonistic manner. This theory was suggested as a result of the way in which photo-receptors are interconnected neurally and also by the fact that it is made more efficient for the HVS to record differences between the responses of cones, rather than each type of cone’s individual response. The opponent color theory suggests that there are three opposing channels called the cone photo-receptors, which are linked together to form three pairs of opposite colors. This theory was first computer modeled for incorporating the color into the LBP texture descriptor by Mäenpää and Pietikäinen [28,49]. It was called Opponent-Color LBP (OC-LBP), and was developed as a joint color-texture operator, thus generalizing the classical LBP, which normally applies to monochrome textures.

Our model is locally based (for each pixel) on nine opposing color pairs and semi-locally, on the set of estimated superpixels of the input image. These nine opposing color pairs are in the RGB (Red—Green—Blue) color space channel: RR, RG, RB, GR, GG, GB, BR, BG and BB (see Section 3.2.2).

**Figure 1:**
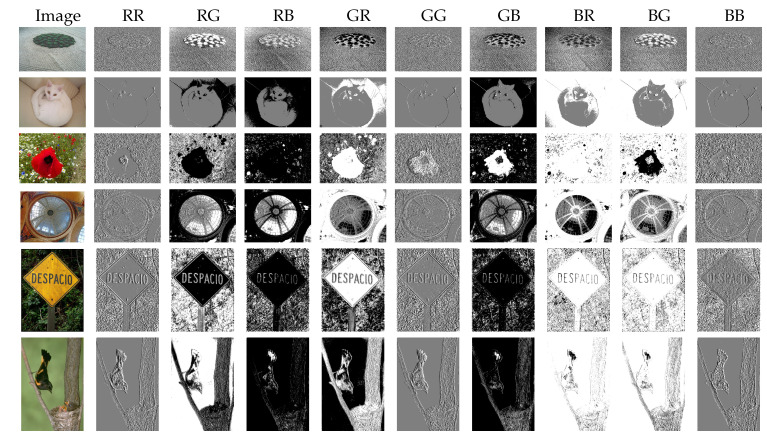
Micro-texture maps given by LTP on the 9 opposing color pairs (for the RGB color space). We can notice that this LTP coding already highlights the salient objects.

The LTP (Local Ternary Patterns) [29] texture characterization method is then applied to each opposing color pair to capture the features of the color micro-textures. At this stage, we obtain nine grayscale texture maps which already highlight the salient objects in the image as can be seen in Figure 1.

**Figure 2:**
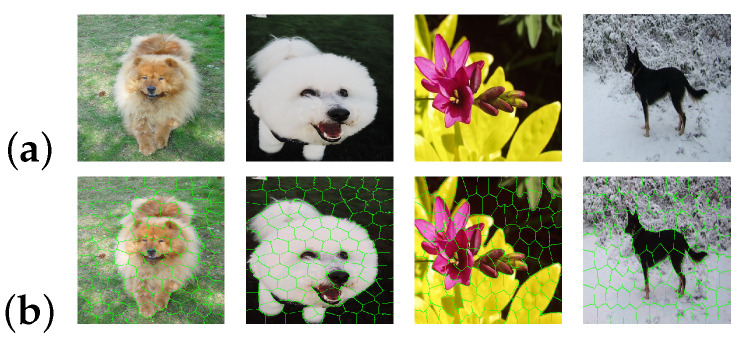
Illustration of SLICO (Simple Linear Iterative Clustering with zero parameter) superpixels bounderies: (**a**) images; (**b**) superpixels.

We then consider each texture map as being composed of micro-textures that can be described by a gray level histogram. As it is not easy to determine in advance the size of each micro-texture in the image, we chose to use adaptive windows for each micro-texture. This is why we use superpixels in our model. To find these superpixels, our model uses the SLICO (Simple Linear Iterative Clustering with zero parameter) superpixel algorithm [33], which is a version of SLIC (Simple Linear Iterative Clustering). The SLICO is a simple, very fast algorithm that produces superpixels, which has the merit of adhering particularly well to the boundaries (see Figure 2) [33]. In addition, the SLICO algorithm (with its default internal parameters), has just one parameter: the number of superpixels desired.

Thus, we characterize each pixel of each texture map by the gray level histogram of the superpixel to which it belongs. We thus obtain a histogram map for each texture map. The nine histogram maps are then concatenated pixel by pixel to have a single histogram map that characterizes the color micro-textures of the image. Each histogram of the latter is then a feature vector for the corresponding pixel.

**Figure 3:**
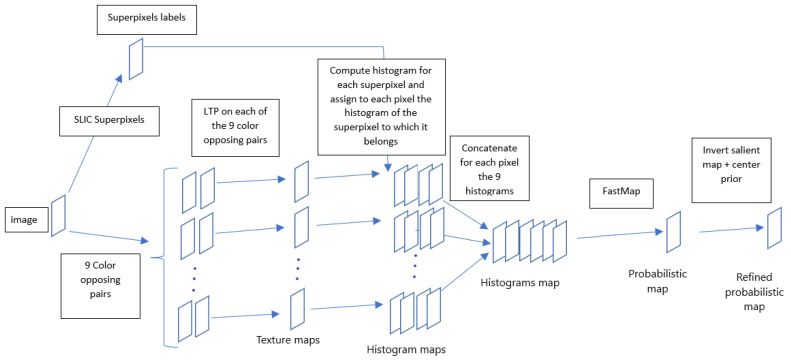
Proposed model steps to obtain the refined probabilistic map from a color space (e.g., RGB: Red–Green–Blue).

The dissimilarity between pixels of the input color image is then given by the dissimilarity between their feature vectors. We quantify this dissimilarity thanks to the FastMap method which has the interesting property of non-linearly reducing in one dimension these feature vectors while preserving the structure in the data. More precisely, the FastMap allows us to find a configuration, in one dimension, that preserves as much as possible all the (Euclidean) distance pairs that initially existed between the different (high dimensional) texture vectors (and that takes into account the non-linear distribution of the set of feature vectors). After normalization between the range 0 and 1, the map estimated by the FastMap produces the Euclidean embedding (in near-linear time) which can be viewed as a *probabilistic* map, i.e., with a set of gray levels with high grayscale values for salient regions and low values for non-salient areas (see Figure 3 for the schematic architecture).

As Borji and Itti [50] stated, almost all saliency approaches use just one color channel. The latter authors also argued that employing just one color space does not always lead to successful outlier detection. Thus, taking into account this argument, we used, in addition to the RGB color space the color spaces HSL, LUV and CMY. Finally, we combine the probabilistic maps obtained from these color spaces to obtain the desired saliency map. To combine the probabilistic maps from the different color spaces used, we reduce for each pixel a vector which is the concatenation of the averages of the values of the superpixel to which this pixel belongs successively in all the color spaces used. In the following section, we describe the different steps in detail.

### 3.2. LTP Texture Characterization on Opposing Color Pairs

#### 3.2.1. Local Ternary Patterns (LTP)

Since LTP (*local ternary patterns*) is a kind of generalization of LBP (*local binary patterns*) [26,51], let us first recall the LBP technique.

The local binary pattern ${\mathrm{LBP}}_{P,R}$ labels each pixel of an image (see Equation (1)).

(1) $${\mathrm{LBP}}_{P,R}\left(x_{c},y_{c}\right)=\sum_{p=0}^{P-1}s\left(g_{p}-g_{c}\right)2^{p},$$

with $\left(x_{c},y_{c}\right)$ being the pixel coordinate and:

$$s\left(z\right)=\left\{\begin{array}{cc} 1 & \;\mathrm{if}\;z\geq 0\\ 0 & \;\mathrm{if}\;z<0, \end{array}\right.$$

where $z\;=\;g_{p}\;-\;g_{c}$.

**Figure 4:**
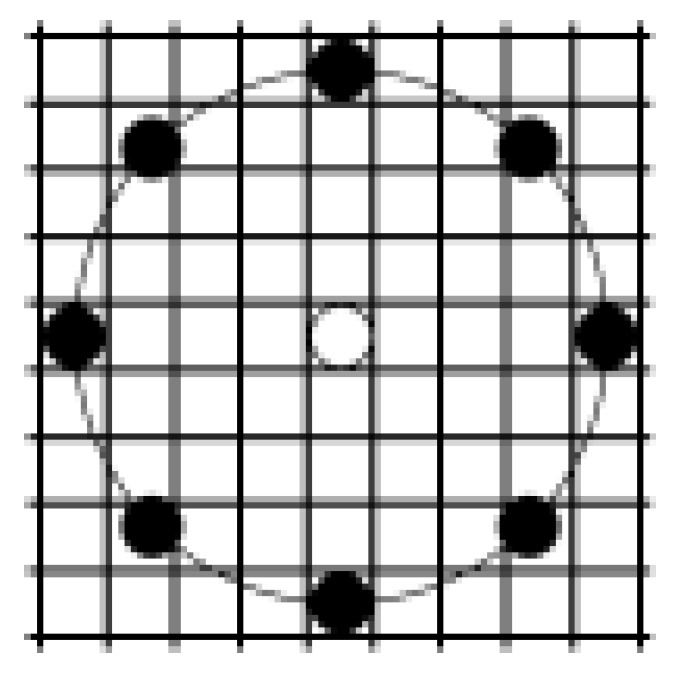
Example of neighborhood (black disks) for a pixel (central white disk) for ${\mathrm{LBP}}_{P,R}$ code computation: in this case P=8, R=4.

The label of a pixel at the position $\left(x_{c},y_{c}\right)$ with $g_{c}$ as gray level is a set of P binary digits obtained by thresholding each gray level value $g_{p}$ of the *p* neighbour located at the distance *R* (see Figure 4) from this pixel by the value of the gray level $g_{c}$ (*p* is one of the *P* chosen neighbors).

**Figure 5:**
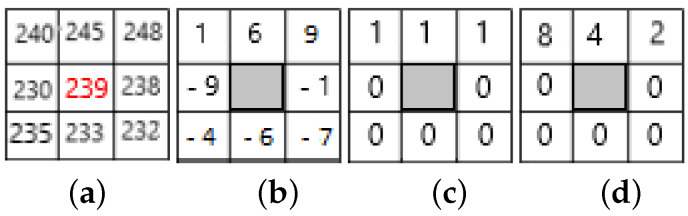
Example of LBP code computation for a pixel: LBP code is 2+4+8=14 in this case. (**a**) pixel neighbourhood; $g_{c}=239$; (**b**) after thresholding; (**c**) pattern: 00001110; (**d**) code = 14.

The set of binary digits obtained constitutes the label of this pixel or its LBP code (see Figure 5).

Once this code is computed for each pixel, the characterization of the texture of the image (within a neighborhood) is approximated by a discrete distribution (histogram) of LBP codes of $2^{P}$ bins.

The LTP (local ternary patterns) [29] is an extension of LBP in which the function s(z) (see Equation (1)) is defined as follows:

$$s\left(z\right)=\left\{\begin{array}{cc} 2 & \;\mathrm{if}\;z\geq t\\ 1 & \;\mathrm{if}\;|z|<t\\ 0 & \;\mathrm{if}\;z\leq -t, \end{array}\right.$$

where $z\;=\;g_{p}\;-\;g_{c}$.

The basic coding of LTP is, thus, expressed as:

(2) $${\mathrm{LTP}}_{P,R}\left(x_{c},y_{c}\right)=\sum_{p=0}^{P-1}s\left(g_{p}-g_{c}\right)3^{p}.$$

**Figure 6:**
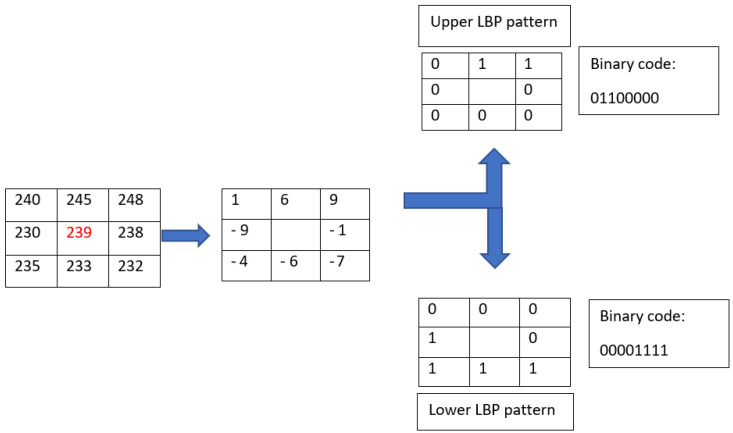
Example of LTP code splitting with threshold t = 3.

Another type of encoding can be obtained by splitting the LTP code into two codes, LBP: Upper LBP code and Lower LBP code (see Figure 6). The LTP histogram is then the concatenation of the histogram of the upper LBP code with that of the lower LBP code [29].

In our model we use the LTP basic coding because we use five neighbors for the central pixel. So the maximum size of the histograms is $3^{5}=243$. In addition, we requantized the histogram with levels/classes of 75 bins for computational reasons (thus greatly reducing the computational time for the next step using the FastMap algorithm while generalizing the feature vector a bit as this operation smoothes the histogram) and we have effectively noticed that this strategy produces slightly better results.

#### 3.2.2. Opposing Color Pairs

**Figure 7:**
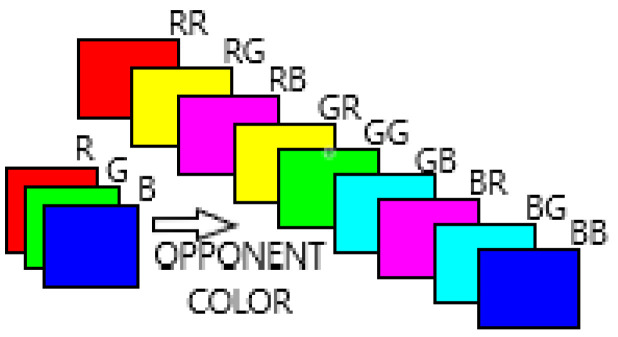
Illustration of color opponent on RGB (Red Green Blue) color space with its 9 opposing color pairs (i.e., RR, RG, RB, GR, GG, GB, BR, BG, BB).

To incorporate the color into the texture description, we rely on the color opponent theory. We thus used the color texture descriptor from Mäenpää and Pietikäinen [28,49], called “Opponent Color LBP”. This one generalizes the classic LBP, which normally applies to grayscale textures. So instead of just one LBP code, one pixel gets a code for every combination of two color channels (i.e., 9 opposing color pair codes). Example for RGB channels: RR (Red-Red), RG (Red-Green), RB (Red-Blue), GR (Green-Red), GG (Green-Green), GB (Green-Blue), BR (Blue-Red), BG (Blue-Green), BB (Blue-Blue) (see Figure 7).

**Figure 8:**
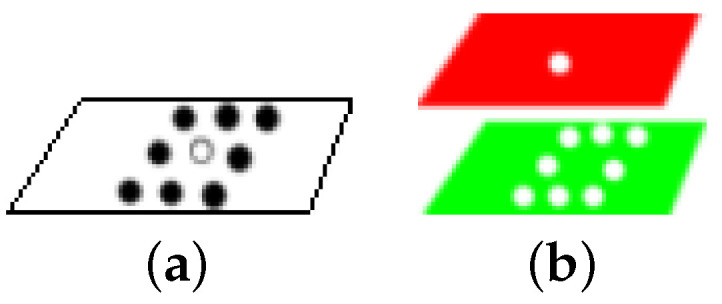
(**a**) Pixel gray LBP code: the code for the central pixel (i.e., white small disk) is computed with respect to his neighbors (i.e., 8 black small disks). (**b**) Pixel opponent color LBP code for RG pair: the central pixel is in the first color channel (red) and the neighbous are picked in the second channel (green).

The central pixel is in the first color channel of the combination and the neighbors are picked in the second color (see Figure 8b).

The histogram that describes the color micro-texture is the concatenation of the histograms obtained from each opposing color pair.

### 3.3. FastMap: Multi-Dimensional Scaling

The FastMap [35] is an algorithm which initially was intended to provide a tool allowing us to find objects similar to a given object, to find pairs of the most similar objects and to visualize distributions of objects in a desired space in order to be able to identify the main structures in the data, once the similarity or dissimilarity function is determined. This tool remains effective even for large collections of datasets, unlike classical multidimensional scaling (classic MDS). The FastMap algorithm matches objects of a certain dimension to points in a *k*-dimensional space while preserving distances between pairs of objects. This representation of objects from a large-dimensional space *n* to a smaller-dimensional space (dimension 1 or 2 or 3) allows the visualization of the structures of the distributions in the data or the acceleration of the search time for queries [35].

As Faloutsos and Lin [35] describe it, the problem solved by FastMap can be represented in two ways. First, FastMap can be seen as a means to represent *N* objects in a *k*-dimensional space, given the distances between the *N* objects, while preserving the distances between pairs of objects. Second, the FastMap algorithm can also be used in reducing dimensionality while preserving distances between pairs of vectors. This amounts to finding, given *N* vectors having *n* features each, *N* vectors in a space of dimension *k*—with n≫k—while preserving the distances between the pairs of vectors. To do this, the objects are considered as points in the original space. The first coordinate axis is the line that connects the objects, called *pivots*. The pivots are chosen so that the distance separating them is at a maximum. Thus, to obtain these pivots, the algorithm follows the steps below:

- choose arbitrarily an object as the second pivot, i.e., the object $O_{b}$;
- choose as the first pivot $O_{a}$, the object furthest from $O_{b}$ according to the used distance;
- replace the second pivot with the furthest object from $O_{a}$, that is, the object $O_{b}$;
- return the objects $O_{a}$ and $O_{b}$ as pivots.

The axis of the pivots thus constitutes the first coordinate axis in the targeted *k*-dimensional space. All the points representing the objects are then projected orthogonally on this axis and in the H hyperplane of n−1 dimensions (perpendicular to the first axis already obtained) connecting the pivot objects $O_{a}$ and $O_{b}$ along the latter axis. The coordinates of a given object $O_{i}$ on the first axis are given by:

(3) $$x_{i}=\frac{d_{a,i}^{2}+d_{a,b}^{2}-d_{b,i}^{2}}{2d_{a,b}},$$

where $d_{a,i}$, $d_{b,i}$ and $d_{a,b}$ are, respectively, the distance between the pivot $O_{a}$ and object $O_{i}$, the distance between the pivot $O_{b}$ and object $O_{i}$, the distance between the pivot $O_{a}$ and the pivot $O_{b}$. The process is repeated up to the desired dimension, each time expressing:

1. the new distance $D^{\prime }\left(\right)$:
  (4) $${\left(D^{\prime }\left(O_{i}^{\prime },O_{j}^{\prime }\right)\right)}^{2}={\left(D\left(O_{i},O_{j}\right)\right)}^{2}-{\left(x_{i}-x_{j}\right)}^{2}.$$
  For simplification,
  $$D^{\prime }\left(O_{i}^{\prime },O_{j}^{\prime }\right)\equiv d_{O_{i}^{\prime },O_{j}^{\prime }}^{\prime },$$
  where $x_{i}$ and $x_{j}$ are the coordinates on the previous axis of respectively the object $O_{i}$ and $O_{j}$.
2. the new pivots $O_{a}^{\prime }$ and $O_{b}^{\prime }$ constituting the new axis,
3. the coordinate of the projected object $O_{i}^{\prime }$ on the new axis:
  (5) $$x_{i}^{\prime }=\frac{d_{a^{\prime },i}^{\prime 2}+d_{a^{\prime },b^{\prime }}^{\prime 2}-d_{b^{\prime },i}^{\prime 2}}{2d_{a^{\prime },b^{\prime }}^{\prime }}.$$

$O_{a^{\prime }}$ and $O_{b^{\prime }}$ are the new pivots according to the new distance expression $D^{\prime }\left(\right)$. The line that connects them is therefore the new axis.

After normalization between the range 0 and 1, the map estimated by the FastMap generates a *probabilistic* map, i.e., with a set of gray levels with high grayscale values for salient regions and low values for non-salient areas. Nevertheless, in some (rare) cases, the map estimated by the FastMap algorithm can possibly present a set of gray levels whose amplitude values would be in completely the opposite direction (i.e., low grayscale values for salient regions and high values for non-salient areas). In order to put this grayscale mapping in the right direction (with high grayscale values associated with salient objects), we simply use the fact that a salient object/region is more likely to appear in the center of the image (or conversely unlikely on the edges of the image). To this end, we compute the Pearson correlation coefficient between the saliency map obtained by the FastMap and a rectangle, with a maximum intensity value and about half the size of the image, and located in the center of the image. If the correlation coefficient is negative (anti-correlation), we invert the signal (i.e., associate to each pixel its complementary gray value).

## 4. Experimental Results

In this section, we present our salient object detection model’s results. In order to obtain the LTP${}_{P,R}$ pixel’s code (LTP code for simplification), we used an adaptive threshold. For a pixel at position $\left(x_{c},y_{c}\right)$ with value $g_{c}$, the threshold for its LTP code is a tenth of the pixel’s value: $t=\frac{g_{c}}{10}$ (see Equation (2)). We chose this threshold because empirically it is this value that has given better results. The number of neighbors P around the pixel on a radius R used to find its LTP code in our model is P=5 and R=1. Thus the maximum value of the LTP code in our case is $3^{5}-1=242$. This makes the maximum size of the histogram characterizing the micro-texture in an opposing color pair to be $3^{5}=243$ which is then requantized with levels/classes of 75 bins (see Section 3.2). The superpixels that we use as adaptive windows to characterize the color micro-textures are obtained thanks to the SLICO (Simple Linear Iterative Clustering with zero parameter) algorithm which is faster and exhibits state-of-the-art boundary adherence. Its only parameter is the number of superpixels desired and is set to 100 in our model (which is also the value recommended by the author of the SLICO algorithm). Finally, we use in the combination to obtain the final saliency map, the color spaces RGB, HSL, LUV and CMY.

We chose, for our experiments, images from public datasets, the most widely used in the salient object detection field [48] such as Extended Complex Scene Saliency Dataset (ECSSD) [52], Microsoft Research Asia 10,000 (MSRA10K) [42,48], DUT-OMRON (Dalian University of Technology—OMRON Corporation) [53], THUR15K [54] and SED2 (Segmentation evaluation database with two salient objects) [55]. The ECSSD contains 1000 natural images and their ground truth. Many of its images are semantically meaningful, but structurally complex for saliency detection [52]. The MSRA10K contains 10,000 images and 10,000 manually obtained binary saliency maps corresponding to their ground truth. DUT-OMRON contains 5168 images and their binary mask. THUR15K is a dataset of images taken from the “Flickr” web site divided into five categories (butterfly, coffee mug, dog jump, giraffe, plane), each of which contains 3000 images. Only 6233 images have ground truths. The images of this dataset represent real world scenes and are considered complex for obtaining salient objects [54]. The SED2 dataset has 100 images and their ground truth.

**Figure 9:**
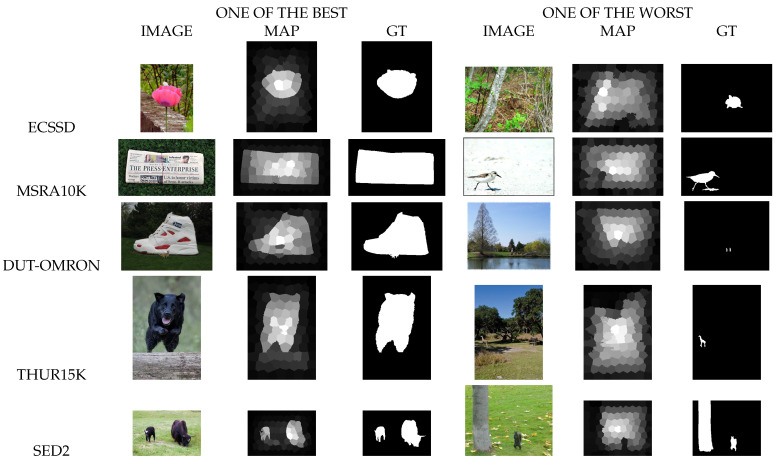
One of the best and one of the worst saliency maps for each dataset used in this work.

We used for the evaluation of our salient object detection model the Mean Absolute Error (MAE), the Mean Squared Error (MSE), the Precision-Recall curve (PR), the $F_{\beta }$ measure curve and the $F_{\beta }$ measure with $\beta^{2}=0.3$. The MSE measure results for ECSSD, MSRA10K, DUT-OMRON, THUR15K and SED2 datasets are shown in Table 1. We compared the MAE (Mean Absolute Error) and the *F*${}_{\beta }$ measure of our model with the 29 state-of-the-art models from Borji et al. [48] and our model outperformed many of them as shown in Table 2. In addition, we can see that our model succeeded to obtain saliency maps close to the ground truth for each of the datasets used although for some images it failed, as shown in Figure 9.

### 4.1. Color Opposing and Colors Combination Impact

**Figure 10:**
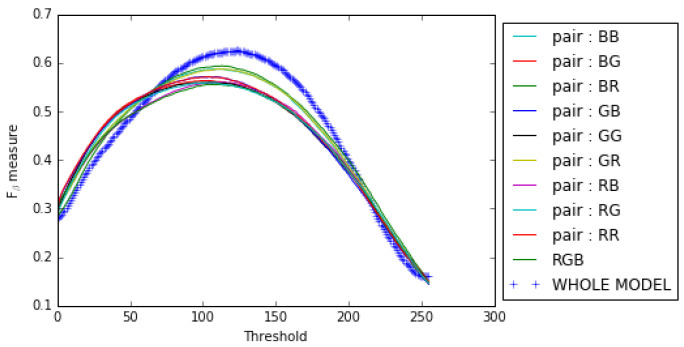
$F_{\beta }$ measure curves for opposing color pairs, RGB color space and the whole model on the ECSSD dataset.

**Figure 11:**
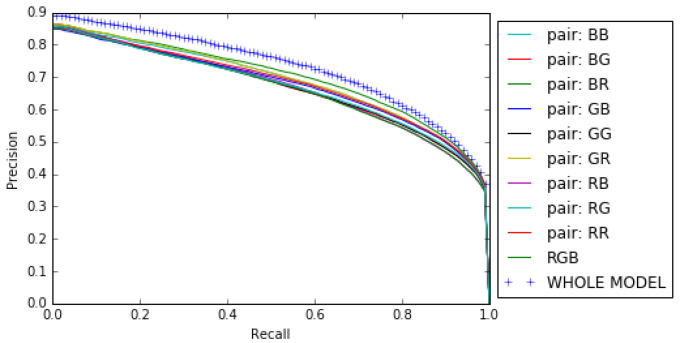
Precision–Recall curves for opposing color pairs, RGB color space and the whole model on the ECSSD dataset.

**Figure 12:**
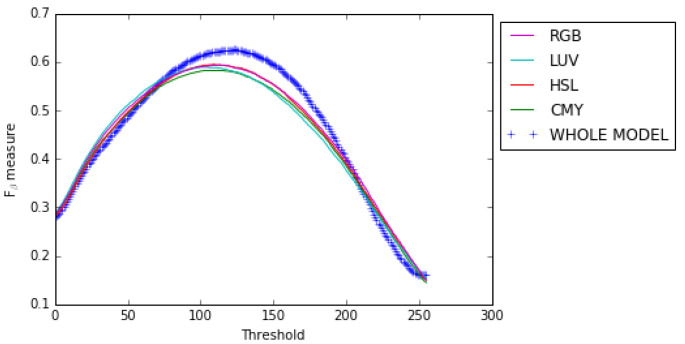
$F_{\beta }$ measure curves for color spaces RGB, HSL, LUV and CMY and the whole model on the ECSSD dataset.

**Figure 13:**
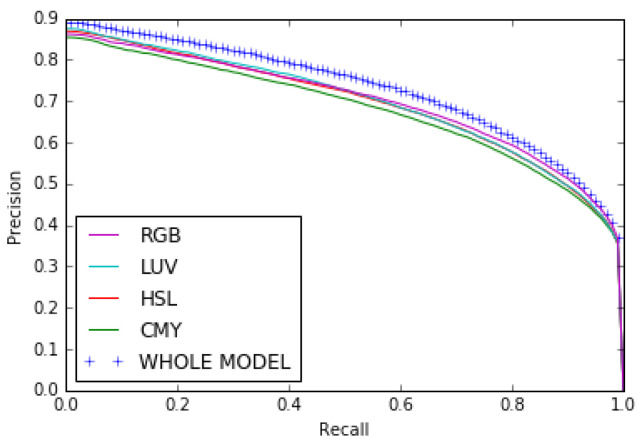
Precision-Recall curves for color spaces RGB, HSL, LUV and CMY and the whole model on the ECSSD dataset.

Our results show that combining the opposing color pairs improves the individual contribution of each pair to the *F*${}_{\beta }$ measure and the Precision-Recall as shown for the RGB color space by the *F*${}_{\beta }$ measure curve (Figure 10) and the Precision–Recall curve (Figure 11). The combination of the color spaces RGB, HSL, LUV and CMY also improves the final result as can be seen from the $F_{\beta }$ measure curve and the precision–recall curve (see Figure 12 and Figure 13).

### 4.2. Comparison with State-of-the-Art Models

In this work, we studied a method that requires no learning basis. Therefore, we did not include machine learning methods in these comparisons.

We compared the MAE (Mean Absolute Error) and *F*${}_{\beta }$ measure of our model with the 29 state-of-the-art models from Borji et al. [48] and our model outperformed many of them as shown in Table 2. Table 3 shows the $F_{\beta }$ measure and Table 4 the Mean Absolute Error (MAE) of our model on ECSSD, MSRA10K, DUT-OMRON, THUR15K and SED2 datasets compared to some state-of-the-art models.

**Table 2:** Number of models among the 29 state-of-the-art models from Borji et al. [48] outperformed by our model on MAE and $F_{\beta }$ measure results.

	ECSSD	MSRA10K	DUT-OMRON	THUR15K	SED2
$F_{\beta }$	21	11	12	17	4
MAE	11	8	6	10	3

#### Comparison with Two State-of-the-Art Models HS and CHS

We have chosen to compare our model to HS [8] and CHS [52] state-of-the-art models because on the one hand they are among the best state-of-the-art models and on the other hand our model has some similarities with these two models. Indeed, our model is a combination of energy-based models MDS and SLICO and is based on the color texture while the two state-of-the-art models are energy based models. Moreover, their energy function is based on a combination of the color and the pixel coordinates.

**Figure 14:**
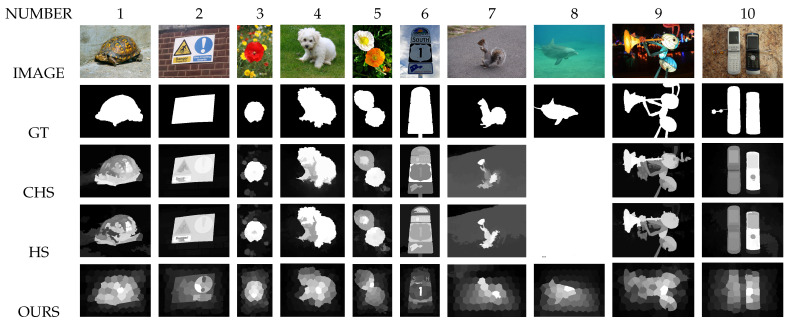
Comparison of some result images for HS [8], CHS [52] and our model. For image number 8, the HS [8] and CHS [52] models find white salient maps (GT: Ground Truth).

First, the visual comparison of some of our saliency maps with those of two state-of-the-art models (“Hierarchical saliency detection”: HS [8] and “Hierarchical image saliency detection on extended CSSD”: CHS [52] models) shows that our saliency maps are of good quality (see Figure 14).

**Figure 15:**
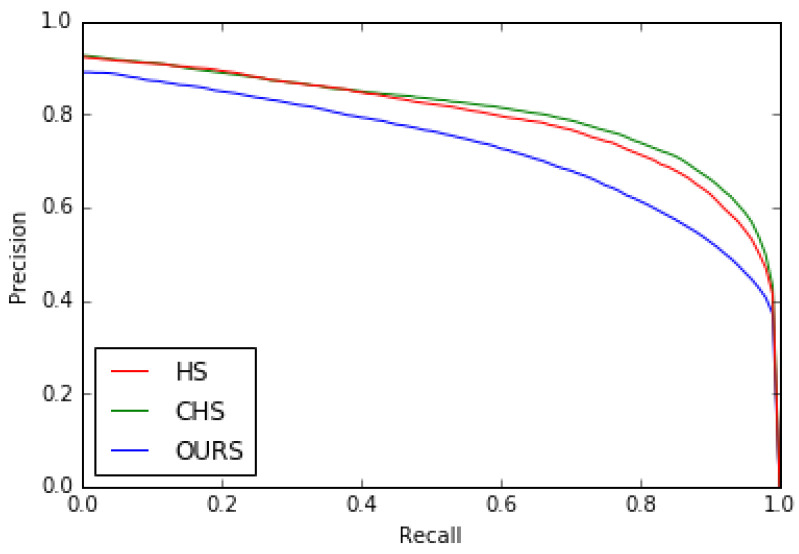
Precision–Recall curves for HS [8], CHS [52] models and ours on the ECSSD dataset.

**Figure 16:**
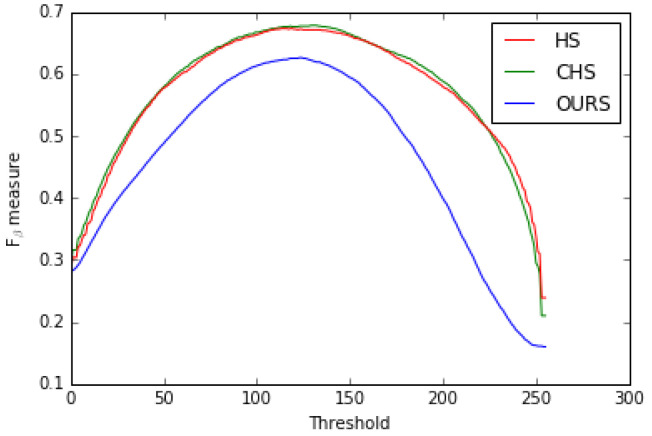
$F_{\beta }$ measure curves for HS [8], CHS [52] models and ours on the ECSSD dataset.

Second, we compared our model with the two state-of-the-art HS [8] and CHS [52] models with respect to the precision-recall, *F*${}_{\beta }$ measure curves (see Figure 15 and Figure 16) and MSE (Mean Squared Error). Table 5 shows that our model outperformed them on the MSE measure.

Thus, our model is better than HS [8] and CHS [52] for the MSE measure while both models are better for the $F_{\beta }$ and Precision–Recall.

Our model also outperformed some of the recent methods for $F_{\beta }$-measure on the ECSSD dataset as shown in Table 6.

## 5. Discussion

**Figure 17:**
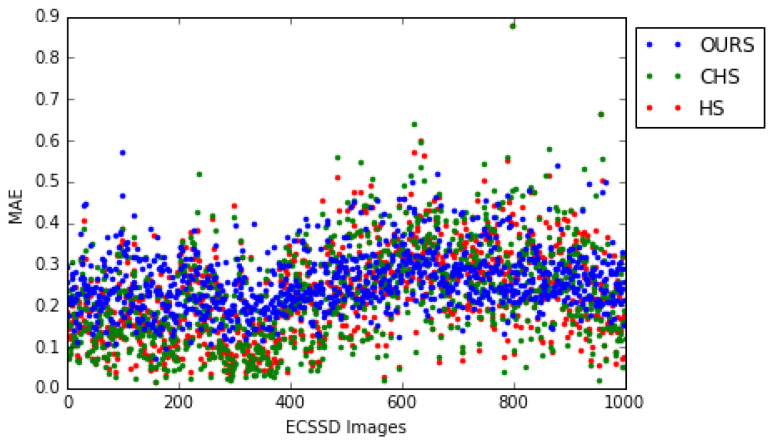
Comparison of the MAE measure dispersion for our model and the HS [8], CHS [52] models on the ECSSD dataset (for MAE, the smaller value is the best).

Our model has less dispersed MAE measures than the HS [8] and CHS [52] models, which are among the best models of the state-of-the-art. This can be observed in Figure 17 but is also shown by the standard deviation which for our model is 0.071 (mean = 0.257), for HS [8] is 0.108 (mean = 0.227), and for CHS [52] is 0.117 (mean = 0.226). For HS [8] the relative error between the two standard deviations is $\frac{(0.108-0.071)\times 100}{0.071}=52.11\%$ while for CHS [52] it is $\frac{(0.117-0.071)\times 100}{0.071}=64.78\%$.

**Figure 18:**
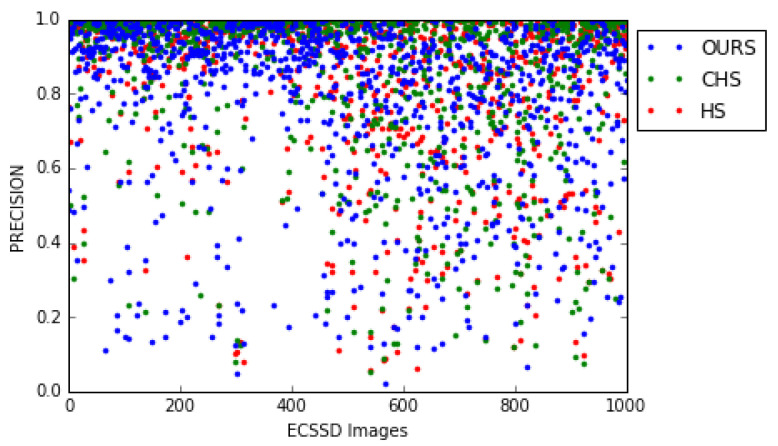
Comparison of the precision measure dispersion for our model and the HS [8], CHS [52] models on the ECSSD dataset.

Our model is stable on new data. Indeed, a model with very few internal parameters is supposed to be more stable for different datasets. We also noticed that nearly 500 first image numbers of the ECSSD dataset are less complex than the rest of the images in this dataset by observing the different measures (see Table 7 and Figure 17 and Figure 18). However, it is clear that the drop in performance over the last 500 images from the ECSSD dataset is less pronounced for our model than for the HS [8] and CHS [52] models (see Table 7). This can be explained by the stability of our model (we used to compute these measures except for MAE a threshold, for each image, which gives the best $F_{\beta }$ measure. It should also be noted that the images are ordered only by their numbers in the ECSSD dataset).

**Figure 19:**
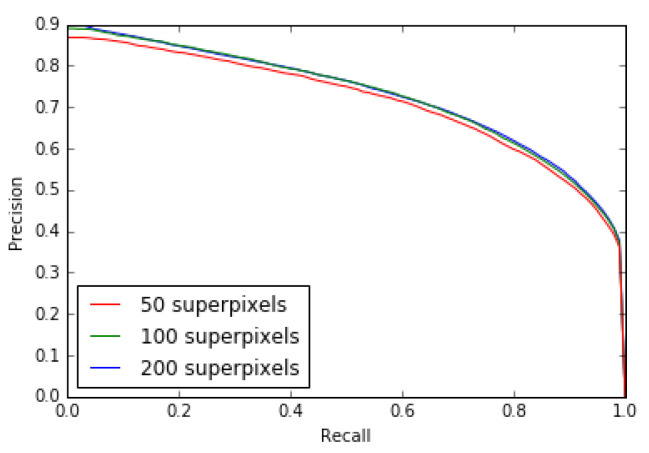
Precision–Recall model’s curves for 50, 100, 200 superpixels (ECSSD dataset).

**Figure 20:**
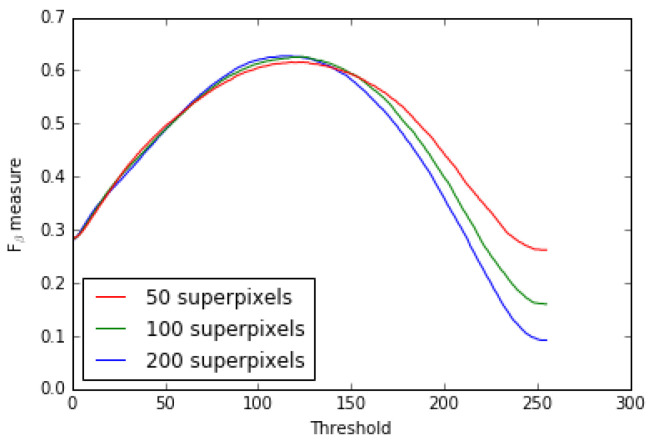
$F_{\beta }$ measure model’s curves for 50, 100, 200 superpixels (ECSSD dataset).

Our model is also relatively stable for an increase or decrease of its unique internal parameter. Indeed, by increasing or decreasing the number of superpixels, which is the only parameter of the SLICO algorithm, we find that there is almost no change in the results as shown by the MAE and $F_{\beta }$ measure (see Table 8) and $F_{\beta }$ measure and precision-recall curves for 50, 100 and 200 superpixels (see Figure 19 and Figure 20).

## 6. Conclusions

In this work, we presented a simple, nearly parameter-free model for the estimation of saliency maps. We tested our model on the complex ECSSD dataset for which the average measures of MAE = 0.257 and *F*${}_{\beta }$ measure = 0.729, and on the MSRA10K dataset. We also tested on THUR15K, which represents real world scenes and is considered complex for obtaining salient objects, and on DUT-OMRON and SED2 datasets.

The novelty of our model is that it only uses the textural feature after incorporating the color information into these textural features thanks to the opposing color pairs theory of a given color space. This is made possible by the LTP (Local Ternary Patterns) texture descriptor which, being an extension of LBP (Local Binary Patterns), inherits its strengths while being less sensitive to noise in uniform regions. Thus, we characterize each pixel of the image by a feature vector given by a color micro-texture obtained thanks to the SLICO superpixel algorithm. In addition, the FastMap algorithm reduces each of these feature vectors to one dimension while taking into account the non-linearities of these vectors and preserving their distances. This means that our saliency map combines local and global approaches in a single approach and does so in almost linear complexity times.

In our model, we used RGB, HSL, LUV and CMY color spaces. Our model is therefore perfectible if we increase the number of color spaces (uncorrelated) to be merged.

As shown by the results we obtained, this strategy generates a model which is very promising, since it is quite different from existing saliency detection methods using the classical color contrast strategy between a region and the other regions of the image and, consequently, it could thus be efficiently combined with these methods for a better performance. Our model can also be parallelized (using the massively parallel processing power of GPUs) by processing each opposing color pair in parallel. In addition, it should be noted that this strategy of integrating color into local textural patterns could also be interesting to study with deep learning techniques or convolutional neural networks (CNNs) to further improve the quality of saliency maps.

## Figures and Tables

**Table 1:** Our model’s MSE measure results for ECSSD, MSRA10K, DUT-OMRON, THUR15K and SED2 datasets (for MSE, the smaller value is the best).

	ECSSD	MSRA10K	DUT-OMRON	THUR15K	SED2
MSE	0.135	0.105	0.130	0.116	0.177

**Table 3:** Our model’s $F_{\beta }$ measure results compared with some state-of-the-art models from Borji et al. [48].

MODELS	ECSSD	MSRA10K	DUT—OMRON	THUR15K	SED2
GR [56]	0.664	0.816	0.599	0.551	0.798
MNP [57]	0.568	0.668	0.467	0.495	0.621
LBI [58]	0.586	0.696	0.482	0.519	0.692
LMLC [59]	0.659	0.801	0.521	0.540	0.653
SVO [60]	0.639	0.789	0.557	0.554	0.744
SWD [61]	0.624	0.689	0.478	0.528	0.548
HC [42]	0.460	0.677	0.382	0.386	0.736
SEG [62]	0.568	0.697	0.516	0.500	0.704
CA [46]	0.515	0.621	0.435	0.458	0.591
FT [63]	0.434	0.635	0.381	0.386	0.715
AC [41]	0.411	0.520	0.354	0.382	0.684
OURS	0.729	0.781	0.531	0.581	0.635

**Table 4:** Our model’s MAE results compared with some state-of-the-art models from Borji et al. [48] (for MAE, the smaller value is the best).

MODELS	ECSSD	MSRA10K	DUT-OMRON	THUR15K	SED2
GR [56]	0.285	0.198	0.259	0.256	0.189
MNP [57]	0.307	0.229	0.272	0.255	0.215
LBI [58]	0.280	0.224	0.249	0.239	0.207
LMLC [59]	0.260	0.163	0.277	0.246	0.269
SVO [60]	0.404	0.331	0.409	0.382	0.348
SWD [61]	0.318	0.267	0.310	0.288	0.296
HC [42]	0.331	0.215	0.310	0.291	0.193
SEG [62]	0.342	0.298	0.337	0.336	0.312
CA [46]	0.310	0.237	0.254	0.248	0.229
FT [63]	0.291	0.235	0.250	0.241	0.206
AC [41]	0.265	0.227	0.190	0.195	0.206
OURS	0.257	0.215	0.267	0.236	0.289

**Table 5:** Our model’s MSE measure results compared with two state-of-the-art HS [8] and CHS [52] models for the ECSSD dataset (for MSE, the smaller value is the best).

	OURS	HS [8]	CHS [52]
MSE	0.135	0.163	0.220

**Table 6:** Our model’s $F_{\beta }$-measure results compared with some of the recent models for the ECSSD dataset.

	OURS	Wu et al. [64]	Yuan et al. [65]	Zhang et al. [66]
$F_{\beta }$-measure	0.729	0.718	0.714	0.725

**Table 7:** Performance drop for Precision and MAE measures with respect to image numbers 0 to 500 (*) and 500 to 1000 (**) of the ECSSD dataset (for MAE, the smaller value is the best).

	Precision			MAE		
	Ours	HS	CHS	Ours	HS	CHS
(*)	0.832	0.919	0.921	0.234	0.176	0.172
(**)	0.737	0.791	0.791	0.279	0.278	0.280
Gap	0.095	0.128	0.130	0.045	0.102	0.108

**Table 8:** Our model’s $F_{\beta }$ measure and MAE results for 50, 100 and 200 superpixels (ECSSD dataset).

Superpixels	50	100	200
$F_{\beta }$ measure	0.722	0.729	0.725
MAE	0.257	0.257	0.257

## Data Availability

The ECSSD dataset is available at 12 February 2022. https://www.cse.cuhk.edu.hk/leojia/projects/hsaliency/dataset.html. The MSRA10K dataset is available at 12 February 2022. https://mmcheng.net/msra10k/. The THUR15K dataset is available at 12 February 2022. https://mmcheng.net/code-data/. The DUT-OMRON dataset is available at 12 February 2022. http://saliencydetection.net/dut-omron/. The SED2 dataset is available at 12 February 2022. https://www.wisdom.weizmann.ac.il/~vision/Seg_Evaluation_DB/dl.html. The HS [8] and CHS [52] models datasets are available at 12 February 2022. https://www.cse.cuhk.edu.hk/leojia/projects/hsaliency/data/ECSSD/our_result_HS.zip and https://www.cse.cuhk.edu.hk/leojia/projects/hsaliency/data/ECSSD/our_result_CHS.zip respectively, available at 12 February 2022.

## Abbreviations

The following abbreviations are used in this manuscript:

HVS	Human Visual System
LTP	Local Ternary Patterns
LBP	Local Binary Patterns
SLICO	Simple Linear Iterative Clustering with zero parameter
SLIC	Simple Linear Iterative Clustering
MDS	Multi-dimensional Scaling
RGB	Red–Green–Blue
HSL	Hue–Saturation–Luminance
CMY	Cyan–Magenta–Yellow
MAE	Mean Absolute Error
ECSSD	Extended Complex Scene Saliency Dataset
MSRA10K	Microsoft Research Asia 10,000 dataset
DUT-OMRON	Dalian University of Technology—OMRON Corporation dataset
SED2	Segmentation evaluation database with 2 salient objects dataset
HS	Hierarchical saliency detection model
CHS	Hierarchical image saliency detection on extended CSSD model
RR	Red-Red
RG	Red-Green
RB	Red-Blue
GR	Green-Red
GG	Green-Green
GB	Green-Blue
BR	Blue-Red
BG	Blue-Green
BB	Blue-Blue
GR [56]	Graph-regularized saliency detection
MNP [57]	Saliency for image manipulation
LBI [58]	Looking beyond the image
LMLC [59]	Bayesian saliency via low and mid level cues
SVO [60]	Fusing generic objectness and visual saliency
SWD [61]	spatially weighted dissimilarity
HC [42]	Histogram-based contrast
SEG [62]	Segmenting salient objects
CA [46]	Context-aware saliency detection
FT [63]	Frequency-tuned salient region detection
AC [41]	Salient region detection and segmentation
